# Success in Plastic Surgery

**Published:** 1893-02

**Authors:** Wm. L. Russell

**Affiliations:** 151 East 50th Street; New York


### *CORRESPONDENCE.*

NEW YORK, January 12, 1893.

### SUCCESS IN PLASTIC SURGERY.

At the last County Society meeting the question of attaining success in gynecological plastic surgery was discussed by Dr. Thomas Addis Emmett. He stated that during the past thirty years he had originated a number of instruments, and had devised a number of operations for plastic operations about the female genitals. Attempts at these operations had now become so general, however, and inexperienced operators were so ready to undertake any condition, that much of his time was taken up in undoing the mischief produced by their work. Plastic surgery was like the game of chess, in which one must have a definite object in view, and know the result of each move. Many had no clear conception of the need in each case while undertaking to supply it. It was now twenty-five years since the profession had been put in possession of the details of the operation for laceration of the cervix, and he questioned now if more harm than good had not resulted. It was a difficult operation to perform, the removal of scar tissue, and the drainage of the angles being all important. The operation for restoration of the anterior vaginal wall had passed into disuse, from the poor results attained because of the faulty manner in which the operation was done. Vesico-vaginal fistulæ were now rarely seen, because of improved midwifery. He had seen fifteen or twenty cases during the thirty-seven years of his connection with the Woman's Hospital. The causes of failure of operations for its cure were, too much traction on the sutures, ignorance of the proper manipulation of Sims' shield, and a faulty adaptation of the urethral catheter. The metallic suture was used only to afford a wide surface for union. Silver wire had been first used for sutures by Sims. It was the best material for suturing the abdominal wall, and after an experience with it for twenty-seven years he had seldom seen hernia follow his operations. Its action was splint-like, producing less constriction of the tissues than silk.

Dr. Mundé said that he did not find silver wire so universally useful as Dr. Emmett did. He used it for the cervix, while for the anterior vaginal wall he thought silk better, and for the posterior wall catgut. In abdominal operations he used catgut and silk-worm gut. Dr. Garrigues believed that the chief element of success was strict antisepsis. Silver wire was the best suture material.

### A NEW ASPIRATOR.

For some reason the proverbial American inventor has been outdone in the matter of aspirators by the Frenchman. Those most commonly used here are the Potain and the Dieulafoy. The Allen pump is also recommended, and is an American invention. Whoever has used it once, however, on an actual case, is likely to wish he had bought some other. The Dieulafoy is by far the best instrument at present in the market. It is very expensive, however, and is very difficult to clean. The Potain is inexpensive, but requires so much muscular exertion to work it, that a new method of creating a vacuum in the bottle will be hailed with delight by whoever has used one.

It is a matter for congratulation, then, that this method has already been found. The possibility of causing a vacuum by absorbing ammonia gas has long been known to chemists, but the practical application of the principle was left for one of the physicians to Demilt Dispensary, Dr. William S. Moore. Dr. Moore has invented an aspirator in which the vacuum is produced by filling a bottle with ammonia gas, and then absorbing it by injecting a small quantity of water. The instrument was exhibited by Dr. Moore at the last meeting of the Section on Practice.

It consists of a vacuum bottle with an opening at the bottom closed by a stopcock. The neck of the bottle is closed by a stopper, through which passes a branching tube. One branch ends in a small glass retort, which is thus suspended over an alcohol lamp fastened to a metallic band which passes around the vacuum bottle. The other branch is connected by a piece of rubber tubing with the aspirating needle.

The mode of operation is to place a small quantity of strong aqua ammonia in the retort and light the lamp, having previously opened the stopcock in the tube leading to the vacuum bottle, and closed that in the tube connecting with the needle. The stopcock at the bottom of the bottle is also to be opened. In two or three minutes the odor of ammonia will be noticed, as the gas, having forced out the air, escapes from the opening at the bottom of the bottle. The light can then be extinguished and the bottle sealed by closing the stopcocks. It is now full of ammonia gas. This gas is so soluble that a drachm of water will absorb a gallon. Its solution in this instance is readily accomplished by introducing a minute quantity of water from a rubber bulb attached to the tube leading to the needle. A vacuum is thus created, and the fluid, passing in through the needle, soon fills the bottle.

The advantages claimed by Dr. Moore for his aspirator are the ease and rapidity with which it can be made to act; its simplicity, which offers very little to get out of order, and no pistons to get dry and loose ; the almost perfect vacuum formed, its cleanliness and sterility, the ammonia gas acting as an antiseptic; and its cheapness, which brings it within the reach of every practitioner.

### FRACTURES IN CHILDREN.

This was the subject at the Section on Practice, a meeting of which was held last evening. The first paper was by Dr. B. F. Curtis, who gave a short history of six cases of fractures which had been overlooked by parents or physicians, or if discovered had been improperly treated. The first case was one of fractured clavicle which had been overlooked by the parents, who supposed it was simply a bruise. In the second case, fracture of the radius and ulna had been overlooked by the family physician, although when the case was seen by Dr. Curtis there was well marked angular deformity. He supposed that it had been originally a green stick fracture. The third case was also a fracture of the radius and ulna. It had been poorly treated, and deformity and complete loss of pronation and supination had resulted. Refracture and new treatment produced a good arm. In the fourth instance a fracture of the radius and ulna had again been overlooked by the physician. The arm had been placed in splints for a week, and was then pronounced recovered. The fifth case was one in which poor treatment had resulted in loss of pronation and supination. In the sixth case a fracture of the internal condyle had not been properly treated, the fragments not having been properly placed, and partial ankylosis had resulted. Resection was necessary in this case. These cases had all been seen within the past two months.

The difficulties offered by the peculiarities of parents and children could not be appreciated by those ignorant of the conditions surrounding the original examination, but such mistakes as those referred to could hardly be justified. The deformity, however, might be slight especially in green-stick fractures, or an accompanying dislocation might complicate the diagnosis. Motion too might be fair. When a child was injured it was well to examine every bone systematically, as was done in an unconscious man who had fallen from a height. The sound limb should be manipulated first, and the child encouraged to move the affected limb. Deformity should be carefully looked for, and local tenderness. The latter point should be left until the last when perhaps the deformity could be quickly reduced at the same time. An anæsthetic might be necessary.

Dr. R. H. M. Dawbarn followed Dr. Curtis with a paper on the treatment of fractures of the thigh in children. He said that the best guide in diagnosis was the relative length of Bryant's base line in the injured as compared with the sound side. The treatment required in any case of fracture of the femur might be classed under one of four heads.

These were as follows:

1. Immobilization.
2. Immobilization with horizontal traction.
3. Immobilization with flexion.
4. Immobilization with flexion and traction.

If the fracture were in the lower two-thirds of the bone, and from direct violence, it would probably be transverse and easy to keep in position. In such a case the treatment would simply consist of immobilization, plaster of Paris or a long side splint being alone required. If, however, a fracture in this location were produced by indirect violence, and therefore oblique, the treatment would come under the second heading and Buck's extension or the elastic extension apparatus of Bryant would be required in addition. Most cases of fracture in children were however transverse. It was in cases in which the fracture was transverse but high up the shaft that the third method of treatment was applicable, the anterior wire splint of Nathan Smith here finding its best application. In oblique fracture in the same situation traction was required in addition, or the fourth method of treatment.

The method of vertical extension was useful in some cases during the first week, allowing as it did a chance for cleanliness and changing of napkins. It was tedious however, and a vulvo-vaginitis sometimes occurred perhaps from the entrance of urine into the vagina. In using plaster of Paris in children two thicknesses only were necessary if the splint was strengthened by narrow strips of tin. It was easy to remove the plaster splint if care were taken to place a piece of narrow tape soaked in oil between each layer of bandage, along the anterior or posterior aspect of the limb. This strip might be very narrow, and prevented the plaster from hardening along its course, so that it cut like putty. He thought it very good practice to administer small doses of phosphorus during the repair of fracture in the form of zinc phosphite. Dr. B. F. Curtis said that he had found the Thomas hip-splint, such as was used in the treatment of hip-joint disease, very useful in some cases.

Dr. Van Arsdale said that fractures of the thigh in very young children were usually transverse. In the new-born they could be treated by flexion on the abdomen. In children of six or eight months however this plan could not be pursued. In such cases he had devised an apparatus which had proved quite satisfactory. It consisted of a hollow pasteboard prism open at each end. This was placed between the abdomen and the flexed thigh, the thin edge toward the joint, and the apparatus, thigh and body were bandaged firmly together.

### NERVINE.

This is a name given to a substance extracted from normal nerve tissue, and being used by Dr. Paul Gibier of the Pasteur Institute for the treatment of epilepsy. He was led to its use by observing the improvement undergone by epileptics under treatment by injection for the prevention of rabies. Dr. Gibier believes that he has seen improvement result in some cases treated by him, but the matter is still *sub judice*.

151 *East 50th Street.* WM. L. RUSSELL.

